# Evaluating Variation in Germination and Growth of Landraces of Barley (*Hordeum vulgare* L.) Under Salinity Stress

**DOI:** 10.3389/fpls.2022.863069

**Published:** 2022-06-16

**Authors:** Jonathan E. Cope, Gareth J. Norton, Timothy S. George, Adrian C. Newton

**Affiliations:** ^1^The James Hutton Institute, Dundee, United Kingdom; ^2^Department of Crop Production Ecology, Swedish University of Agricultural Sciences, Uppsala, Sweden; ^3^School of Biological Sciences, University of Aberdeen, Aberdeen, United Kingdom

**Keywords:** barley landraces, *Hordeum vulgare*, Bere barley, genetic diversity, salinity tolerance

## Abstract

Ongoing climate change is resulting in increasing areas of salinity affected soils, rising saline groundwater and droughts resulting in irrigation with brackish water. This leads to increased salinity stress in crops that are already grown on marginal agricultural lands, such as barley. Tolerance to salinity stress is limited in the elite barley cultivar pools, but landraces of barley hold potential sources of tolerance due to their continuous selection on marginal lands. This study analyzed 140 heritage cultivars and landrace lines of barley, including 37 Scottish Bere lines that were selected from coastal regions, to screen for tolerance to salinity stress. Tolerance to salinity stress was screened by looking at the germination speed and the early root growth during germination, and the pre-maturity biomass accumulation during early growth stages. Results showed that most lines increased germination time, and decreased shoot biomass and early root growth with greater salinity stress. Elite cultivars showed increased response to the salinity, compared to the landrace lines. Individual Bere and landrace lines showed little to no effect of increased salinity in one or more experiments, one line showed high salinity tolerance in all experiments—Bere 49 A 27 Shetland. A Genome Wide Association Screening identified a number of genomic regions associated with increased tolerance to salinity stress. Two chromosomal regions were found, one associated with shoot biomass on 5HL, and another associated with early root growth, in each of the salinities, on 3HS. Within these regions a number of promising candidate genes were identified. Further analysis of these new regions and candidate genes should be undertaken, along with field trials, to identify targets for future breeding for salinity tolerance.

## Introduction

Saline soils are soils that have accumulated salt beyond a critical level, while sodic soils are soils with high levels of exchangeable sodium ions and can be accompanied with excess salt (Osman, 2018). The FAO (2015) estimate that 6.5% of land around the world are salt-affected, of which over half are sodic soils. One cause of salinity in soil is the practice of irrigation with brackish waters, causing a steady build-up of salt through evaporation (Ayars et al., 1993; Umali, 1993; Wei et al., 2018). Salt build-up in non-irrigated soils is called dryland salinity, occurring through wind dispersal or rising saline groundwater tables leaving deposits of salt that cannot be washed away by leaching and runoff. Dryland salinity can be due to natural fluctuations (primary salinity), or man-made through vegetation clearing (secondary salinity); (Pannell and Ewing, 2006; McFarlane et al., 2016; Akter, 2017). Coastal incursion of saline water into groundwater is also a problem, particularly in areas prone to sea-level rise. All these causes of salinity are likely to increase with man-made climate change due to the increase in need for irrigation, impacts on natural vegetation and rising sea levels, respectively (Paranychianakis and Chartzoulakis, 2005; Rengasamy, 2006; Barbieri et al., 2020) particularly in arid regions (Corwin, 2021).

High levels of soil salinity have a dual negative effect in plants, like most elements there is an ionic effect, but in addition there is an osmotic effect in soils that decreases the ability for roots to take up water, simulating aspects of osmotic stresses found due to drought (Munns and Tester, 2008). The osmotic stress is rapid, occurring within minutes, and results in the decreased growth of new shoots, along with slower emergence of leaves and lateral buds. Ionic toxicity is caused when salt accumulates in the plant tissue to toxic levels. This is a slower effect, occurring over multiple days, and causes an increased rate of senescence in the older leaves (Munns, 2002; Munns and Tester, 2008; Roy et al., 2014). Ionic toxicity plays a role in the interaction with other nutrients due to specific ion toxicity. This is where increased levels of ions, such as Na^+^, compete with essential nutrients for uptake and metabolism in the plant, potentially causing a deficiency in nutrients such as P, N, Ca, and K (Parihar et al., 2015). Saline and sodic soils are also associated with limited micronutrient solubility, resulting in an interaction that increases the deficiency of micronutrients such as Cu, Fe, Zn, Mo, and Mn (Grattan and Grieve, 1998), with the application of these micronutrients helping alleviate some of the salinity stress (Pandya et al., 2005; Pérez-Labrada et al., 2019; Noreen et al., 2021). As a result of the different types of stresses produced by saline conditions, there are different mechanisms of resistance. The three broad categories of resistance are: the exclusion of Na ions from the leaf tissue to prevent ionic stress, the tolerance to osmotic stress, and the tolerance to ionic stress, such as Na ions build-up in the leaf tissue, through methods such as compartmentalization into the vacuole (Munns and Tester, 2008).

Due to lack of economically practical screening methods, and the fact that salinity tolerance is a highly complex trait composed of resistance to both ionic and osmotic stress, conventional breeding for salinity tolerance in barley is limited. Additional problems arise in the differential result, and possibly mechanism, of resistance between salinity tolerance in seedling and germination, and between hydroponic and soil systems.

The continuous growth of landraces on marginal soils, can potentially be a valuable source of genetic material for tolerances against abiotic stresses. One of the most common abiotic constraints is drought, affecting large regions of low rainfall areas that depend on rain-fed water application, in both more and less economically developed countries. Landraces have been a large source of drought tolerance in arid regions such as Ethiopia (Abera, 2009), Namibia (Ben Naceur et al., 2012), Tunisia (Dbira et al., 2018), and particularly in the region of the fertile crescent such as Syria (Grando et al., 2001), Iran (Pour Aboughadareh et al., 2013), and Jordan (Haddadin, 2015). Prolonged drought stress events in otherwise water adequate environments is an alternative, but related, water deficient stress; landraces from the Mediterranean region have shown a tolerance that could be utilized (Comadran et al., 2007). Like drought, salinity stress can manifest as an osmotic stress, and thus likely has overlapping mechanisms of tolerance. Similarly, landraces that express tolerance to salinity stress have been specifically identified in populations from Morocco (El Madidi et al., 2004), Oman (Jaradat et al., 2004), Egypt (Elsawy et al., 2018), and Syria (Kalaji et al., 2011), along with an Algerian landrace (Karakousis et al., 2003; Hayes and Reid, 2004; Widodo et al., 2009).

The Scottish landrace ‘Bere’ has been grown on predominately marginal lands for, at least, the last half millennia, and currently is grown on the highlands and islands of Scotland (Jarman, 1996), mainly in soils in coastal zones. Bere barley is generally referred to as a single variety and is often shown to have different, sometimes conflicting, phenotypes (Wright et al., 2002; Scholten et al., 2004; Newman and Newman, 2006; Martin et al., 2008). Though it has been shown by Southworth (2007) that Bere barley contains large diversity. In that study, 29 microsatellite markers showed that there is significant genetic variance between the three island groups of the Shetland, Orkney, and Western Isles and this genetic variance was further validated using a 50 K SNP array (Schmidt et al., 2019). It was suggested that this clustering is due to the lack of historical seed trade between the island groups, but also suggested that it could be due to adaptation to the differing environments between the islands. This diversity manifests as differing phenotypic traits with regards to both biotic and abiotic stresses, potentially differing due to unique nutrient deficiencies and toxicities found in the different environments of the islands farming area. This collection had not previously been screened for salt tolerance and thus the potential of this genetic material remained undiscovered.

Recent advancements in genotyping have allowed for the identification of quantitative trait loci (QTLs) and the use of marker assisted breeding (Mano and Takeda, 1997; Tavakkoli et al., 2010; Zhou et al., 2012; Ashraf and Foolad, 2013). These tools have the potential to identify regions associated with salinity tolerance from sources such as tolerant landraces (Newton et al., 2010; Allel et al., 2016; Dwivedi et al., 2016). For example, QTLs were found in a Chinese landrace by Xu et al. (2012), as well as the potential loci found in wild varieties of barley (Nevo and Chen, 2010), both showing associations with increased salinity tolerance in barley. To date (Kumar et al., 2020), the major QTLs for salinity tolerance have been found on 1HL (Rivandi et al., 2011), 2HS (Xu et al., 2012; Gill et al., 2019), 7HS (Shavrukov et al., 2013). Despite this, very few genes have been incorporated into commercial breeding lines, and salinity stress still remains a global problem (Asif et al., 2019). This, together with the complexities of salinity stress, highlights the need to identify a number of different tolerance mechanisms that can be used in breeding salinity tolerant lines for climate resistant crops. Additionally, tolerances at different stages do not necessarily correlate, with temperate cereal crops showing higher sensitivity to salinity during emergence and early seedling growth (Maas and Hoffman, 1977).

The aims of this study were to identify Bere and other landrace lines that are able to maintain germination and biomass when grown in saline growth media. This data was used along with the genotypic data to identify genomic regions associated with this trait using genome-wide association study (GWAS). These regions were then searched for encoded candidate genes that have a putative function associated with salinity tolerance. The goal of this project was to identify candidate genes for future characterization and possible incorporation into commercial breeding programs to breed for barley crops that are better able to tolerate increasing saline conditions as a result of climate change.

## Materials and Methods

### Barley Material Preparation

A total of 140 lines (Supplementary Table 1) from The James Hutton Institute (Dundee, United Kingdom) Heritage Spring Barley Landrace Collection (JHI-SBLC) were selected, with original seed selections predominantly originating from the JIC-GRU (John Innes Centre Germplasm Resources Unit) or SASA (Science and Advice for Scottish Agriculture) collections. This contained landraces, including 37 Bere lines, and other heritage cultivars. The lines were multiplied in glasshouse conditions as outlined in Cope et al. (2020). The lines were also categorized into two sub-categories of; Bere lines consisting of all the lines labeled as Beres, and “other landraces” consisting of the other landrace lines and the old varieties included in the collection. These categories were used to compare against each other and against the elite cultivars that were grouped into a third sub-category.

### Shoot Biomass Growth Screen

For practical reasons due to the scale of this experiment the four replicates of the 140 landrace lines in three composts with differing NaCl levels were split into eight individual trials. Each trial was comprised of one replicate of half the landrace lines, a total of 70, along with the five elite cultivars (Concerto, Optic, Propino, Wagon, and Westminster), grown in all three compost concentrations (0, 50, and 100 mmol/kg NaCl). Each line was used in a total of four trials, the elite cultivars were used in all eight. The trials were designed so that each line occurs in the same trial with most other lines, with nearly 60% of all line pairs occurring.

For each experimental trial the 70 lines and the five elite cultivars, 10 seeds were germinated on water agar, for 3 days at room temperature. Nine batches of universal compost—made as outlined in Cope et al. (2020)—were weighed out to 15 kg each and amended with three salt concentrations of 0, 50, and 100 mmol/kg NaCl (0, 43.8, and 87.7 g per batch, respectively). The 45 kg of each compost concentration was then distributed into 75 12.7 cm × 12.7 cm pots (1,200 cc). One seedling from each line/cultivar was then transplanted to one pot of each of the three salt concentrations of compost, randomized and arranged into five blocks of 45 pots.

Trials were grown in a climate-controlled glasshouse with a day/night temperature of 15/18°C and supplementary lighting (at 200 μmol quanta m^–2^ s^–1^) provided when light intensity was less than 200 W m^–2^ and shading when above 450 Wm^–2^ to give a day length of 16 h [as outlined in Cope et al. (2021)]. Relative humidity was not controlled. Plants were grown between Aug-Nov 2016, approximately 2 weeks apart in trial number order. After approximately 70 days of growth the individual plants were harvested by cutting the shoots at the surface of the soil. Each plant had measurements taken of its longest tiller for length, number of tillers, the number of ears (if heading), and then was weighed and dried in a drying oven at 50°C for 4 days.

### Germination and Early Root Growth Screen

Water agar with NaCl concentrations of 0, 100, and 200 mmol/l was made by mixing 0, 5.8, and 11.6 g of NaCl to one liter, respectively. This was then spread over fifteen 140 mm Petri dishes and allowed to cool.

A selection of 135 lines (listed in Supplementary Table 1) were arranged over the 15 plates for each of the three salt concentrations, using one seed per line. The seeds were arranged near the edge of the dish 36° apart, with nine lines per dish. One of the five elite cultivars was placed remaining space in each, resulting in three times as many reps of the elite cultivars than the other lines. These were kept in a growth cabinet at 17°C, covered to exclude light, for 7 days. The germination and coleoptile emergence status was recorded every 12 h. Pictures of the root growth were taken every 24 h from day 2, and measured using ImageJ software. This was repeated five times to get replication of the lines.

### Genotypic Data

Germination, DNA isolation, genotyping, and analysis were carried out as previously to obtain the genotypic data (Cope et al., 2020). The genotypic data was processed by removing the markers that had a low call rate (< 80%) or low minor allele frequency (< 10%), along with the genotypic lines that had a low rate of marker return (< 80%) or large heterozygosity (False Discovery Rate < 10%). The statistical program R (R Core Team, 2013), with the GenABEL package (Aulchenko et al., 2007), was used to perform GWAS using a Mixed Linear Model (MLM) approach or EIGENSTRAT approach controlling for population structure and relatedness, as outlined in Yu et al. (2006) and Zhang et al. (2010), with the dry weight data from the biomass growth screen, as well as the radicle emergence and root growth data from the germination and early root growth screen. Quality controls for the GWAS were performed using quantile-quantile (QQ) plots. The region of the QTL was identified by a LOD drop of greater than two, or the range between the first and last marker in the peak ± 0.5 Mbp, taking whichever was the greater range. This region was then searched using the Barlex database (with the Morex v2 Gene Models) for genes contained within (Colmsee et al., 2015).

### Statistical Analysis

The raw data from the biomass measurements was fitted to a slope, using the three salt concentrations from each replicate for each line/cultivar to create four replicate slope scores, with the 0 mmol/kg data points being used as y intercepts. This was done for each of the measurements; dry weight, wet weight, height, tiller number, and head number. This was repeated to get a slope as a percentage of the control. An unbalanced ANOVA was performed in the statistical program Genstat 18*^th^* edition (VSN International, United Kingdom), using line/cultivar (or sub-category) as the treatment and replicate as the blocking factor.

The radicle and coleoptile emergence times from the germination and early root growth screen were adjusted to account for the difference in set up time. These variates were used in an ANOVA in the statistical program Genstat; using line/cultivar (or sub-category), and salt concentration as treatments, with replicate as the blocking factor.

Root growth progression was analyzed using root length transformed by (+ 1)log_10_ as the variate. This was analyzed with a repeated measurement ANOVA in Genstat, using line/cultivar (or sub-category) and salt concentration as treatments, individual SeedID as the blocking factor, and day as the time point. A line was fitted to the averages of each of the lines/cultivars over the days to get the root growth rate for each salt concentration.

## Results

### Shoot Biomass Growth

**FIGURE 1:**
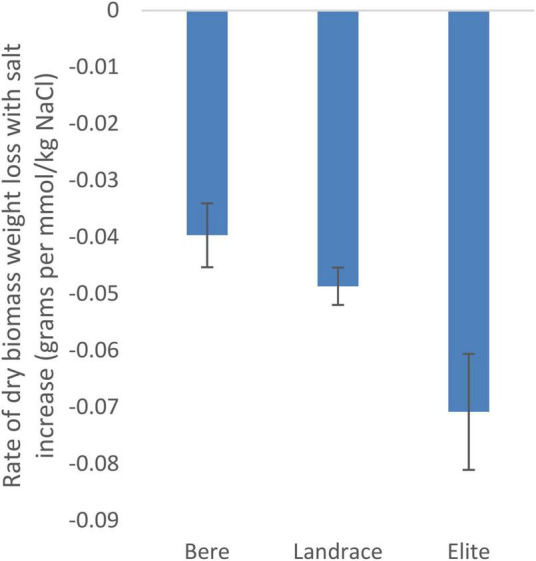
The salinity tolerance as measured by the average change in dry weight with increasing salt concentration for 146 lines/cultivars of barley divided into three groups, Bere, Other landraces, and elites (*n* = 37, *n* = 104, and *n* = 5, respectively). Complete resistance is represented as 0, with negative measurements showing susceptibility. Error bars represent the standard errors in positive and negative directions.

Using the slope integer from the fitted linear equation there was a significant difference between the three sub-categories (Bere, other landraces, and elite lines) for dry weight (*p* = 0.028; Supplementary Table 2) and tiller number (*p* = 0.007; Supplementary Table 3), but not for fresh weight and height (Supplementary Tables 4, 5, respectively). The rate of dry biomass weight loss with salt increase (Figure 1), was approximately double in the elite cultivars, at 0.07 g per mmol/kg of NaCl (or 0.39% of the control per mmol/kg), than that of the Bere lines at 0.04 g per mmol/kg of NaCl (or 0.17% per mmol/kg).

**FIGURE 2:**
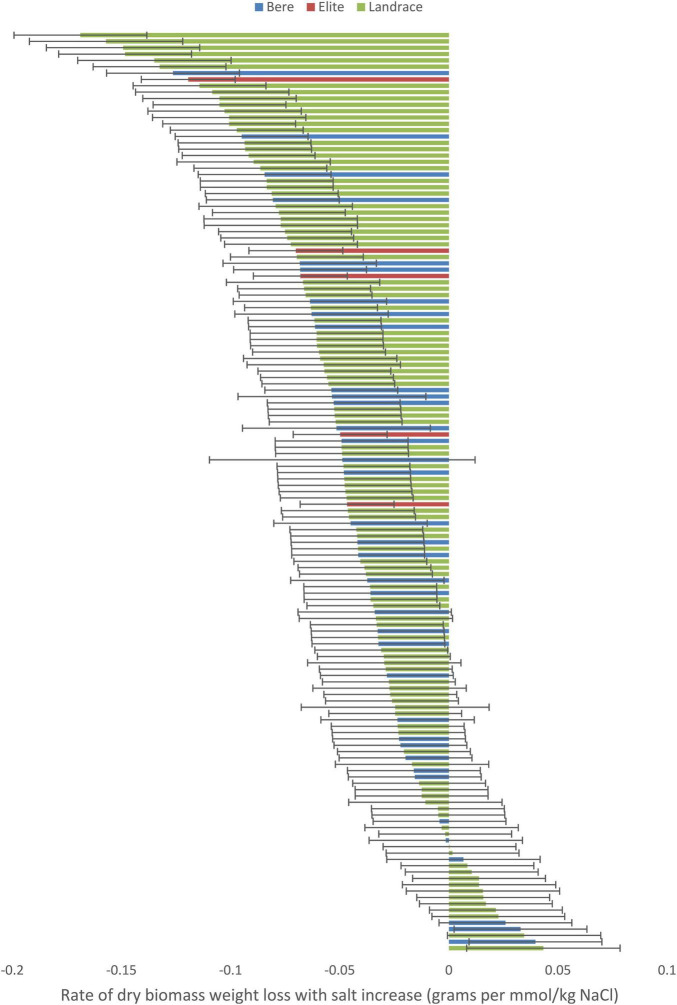
The salinity tolerance measured by the average change in dry weight with increasing salt concentration for 140 lines and 5 elite cultivars of barley grown for approximately 70 days in universal compost. Complete resistance is represented as 0, with negative measurements showing susceptibility, and positive measurements showing salinity preference. Error bars represent the standard errors in positive and negative directions.

When the data with the slope integer fitted was analyzed for, a significant difference was found between the individual lines/cultivars for both the dry weight (*p* < 0.001; Supplementary Table 6) and fresh weight (*p* = 0.011; Supplementary Table 7) data, and this significance was maintained when looking at the slope data calculated as a percentage of the control (*p* = 0.040 and 0.010, respectively, Supplementary Tables 8, 9). No significant difference were found within either the Height nor tiller number data. Three lines showed a significant increase in dry weight with increased salt levels: Prize Prolific-196, Bere-118, and Bere 49 A 27 Shetland (Figure 2), with increases of 0.043, 0.040, and 0.032 g per mmol/kg, respectively (and increases of 0.35, 0.45, and 0.4% per mmol/kg, respectively). Fifteen lines had large weight reductions of 0.1 g per mmol/kg (and over 0.5% per mmol/kg), including elite cultivar Wagon, and Bere 39 A 16 Berneray.

#### Genome-Wide Association Study Analysis

From the 37,242 markers used, 10,593 were removed as having low (< 10%) minor allele frequency and 30 due to a low call rate. Of the 140 lines used, 13 were excluded because their heterozygosity was too high and nine due to being identical by state (IBS).

**FIGURE 3:**
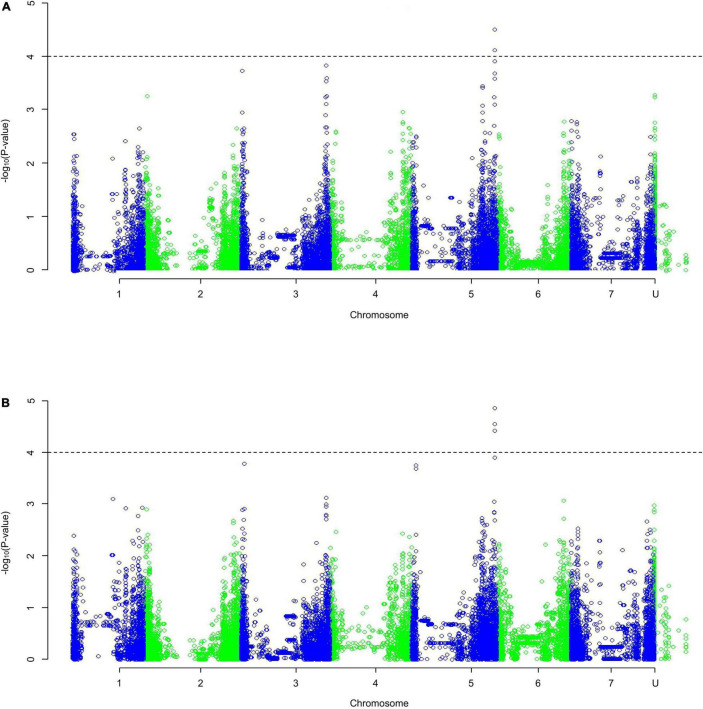
A Manhattan plot of a Genome-Wide Association Study undertaken using a Mixed Linear Model on the average change in dry weight with increasing salt concentration as **(A)** weight, **(B)** percentage of the average control weight; data generated using an ANOVA. Depressions in marker significance observed in the center of each chromosome are due to reduced marker density around the centromere of the physical map.

The QQ plots (Supplementary Figure 1) showed that the MLM approach had the smallest deviation from the expected null distribution when looking at dry weight. Both the slope integer datasets [Figure 3; weight (a) and percentage (b)] identified one region of significance on the distal end of chromosome 5HL. Only five markers with p-values of < 0.0001 in both analyses were identified (others were identified in only one analysis), found in the distal end of 5HL; three at 651.49–651.52 Mb and two at 651.20 Mb. The five markers identified in 5HL were amongst the largest negative effects, with decreases of 0.023–0.028 g per mmol/kg (0.155–0.185% per mmol/kg).

Within the region 651.10–651.60 Mb of 5HL there were a total of 29 associated genes, of which four were identified as candidate genes (listed in Table 1) that encode for: a Lysine-specific demethylase REF6, an Actin 7, a Ferredoxin 3, and an Acyl-CoA-binding domain-containing protein 4, the latter of which contained the two most significant markers for data sets, while the former of which is a low confidence gene.

**TABLE 1:** Candidate genes identified in relation to salinity tolerance in regards to biomass growth, with the chromosome and position on the physical map listed.

Gene Name	Chr	Position	Annotation
HORVU5Hr1G117860.5	5HL	651.33	Lysine-specific demethylase REF6
HORVU5Hr1G117900.1	5HL	651.48	Actin 7
HORVU5Hr1G117910.3	5HL	651.49	Ferredoxin 3
HORVU5Hr1G117970.2	5HL	651.52	Acyl-CoA-binding domain-containing protein 4

Of these, actin was the most common annotation representing 0.23% of the 73,586 genes listed in BARLEX the barley genome explorer (Colmsee et al., 2015), at 172 genes genome-wide. The remaining annotations—Lysine-specific demethylases, ferredoxins, and Acyl-CoA-binding proteins—are less common representing ≤ 0.08% of the genome each, at 59, 30, and 23 genes, respectively.

### Germination and Early Root Growth

#### Emergence Speed Analysis

Comparison of the sub-categories showed a significant difference among the: concentration (*p* < 0.001), sub-category (*p* = 0.002), and interaction (*p* = 0.002) for the radicle emergence time (Supplementary Table 10). Coleoptile emergence time was only significantly different among the concentrations (*p* < 0.001) and sub-categories (*p* = 0.005), but not for the interaction (*p* = 0.106; Supplementary Table 11). The elite cultivars had the quickest radicle emergence for both salt concentrations, with an increased germination time of 10 and 41% for the 100 and 200 mmol/l salt concentrations, respectively. The Bere lines had an average increase in germination time of 22 and 64%, respectively (Supplementary Figure 2).

**FIGURE 4:**
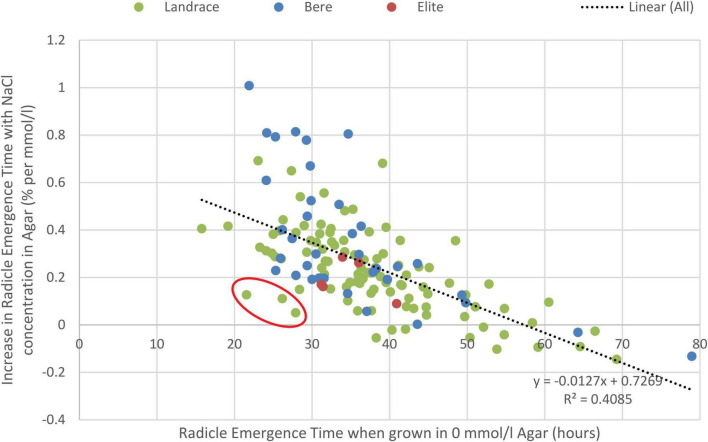
Correlation between the delay in germination due to salinity (measure by percentage increase in radicle emergence time with increased salt concentrations) and the control germination rate of the experiment (measured as the radicle emergence time in control conditions). The line of best fit along with the coefficient of determination (*R*^2^) value is given; *p* < 0.001. Circled in red are Lawina, HSX07-26, and Nepal 92 BN-1, lines identified as both fast to germinate and able to maintain most of this speed with increasing salt concentrations.

The ungrouped data similarly showed significant differences (p-values < 0.001) between the: lines/cultivars, salt concentrations, and interaction, seen for both the radicle (Supplementary Table 12) and coleoptile emergence time (Supplementary Table 13). Lines/cultivars with longer radicle emergence time in control conditions were less effected by the increase in salt concentration (Figure 4). This allowed for identification of lines that are both fast germinators and maintain this speed in increasing salt concentrations, this includes the landrace lines Lawina, HSX07-26, and Nepal 92 BN-1 that were amongst the fastest emerging lines in the highest salt concentration, germinating in 21–28 h in the control and 28–33 h in the highest salt levels. Comparatively, the fastest elite cultivar was Concerto with a germination time of 42 h in the highest salt concentration and an increase of 0.16% per mmol/l.

#### Root Growth Analysis

Sub-category data of the transformed root growth showed a significant difference among sub-categories (*p* < 0.001), and salt concentrations (*p* < 0.001), but not the interaction of these two treatments (*p* = 0.210), until the time factor was included (*p* = 0.031; Supplementary Table 14). Bere lines showed an increased root elongation rate for all salt concentrations compared to the elite cultivars (Supplementary Figure 3).

Individual lines/cultivars similarly showed a significant difference among: sub-categories, salt concentrations, and the interaction of these two treatments, including when the time factor was accounted for (*p*-values < 0.001; Supplementary Table 15).

Floye and Bere 49 A 27 Shetland had amongst the highest growth rate in both salt concentrations, the latter of which had amongst the lowest growth rates in the control (Supplementary Figure 4). The growth rates of these lines were 4.1–4.4 and 2.6—.8 mm/day in 100 and mmol/l NaCl, respectively, compared to the fastest growing elite cultivar, Optic, that had growth rates of 1.8 and 1.1, respectively.

#### Genome-Wide Association Study Analysis

From the 37,242 markers used, 10,795 were removed as having low minor allele frequency and a further 32 because of a low call rate. Of the 135 lines used 13 were excluded because their heterozygosity was too high, and a further nine due to being identical by state (IBS).

The QQ plots for the radicle emergence data showed that for all three salt concentrations (Supplementary Figure 5) the EIGENSTRAT approach had the lowest deviation from the expected null distribution. The Manhattan plots (Supplementary Figure 6) show no significant association peaks. Analysis of the percentage change in radicle emergence time was displayed in a MLM Manhattan plot (Supplementary Figure 7), selected based on the QQ plot as above (Supplementary Figure 8), similarly this plot showed no significance. What was indicated was a peak toward the center of chromosome 5HL, this was in the same position as that found in the GWAS of the salinity tolerance.

**FIGURE 5:**
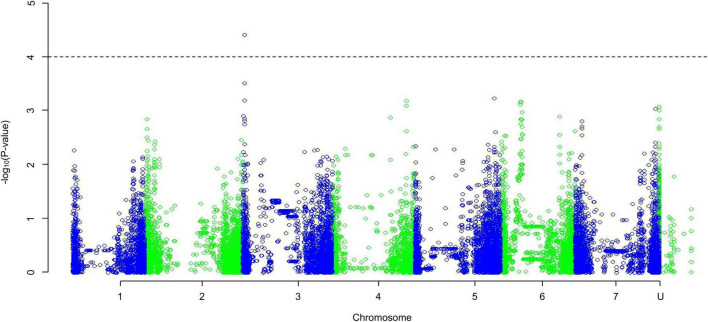
A Manhattan plot of a Genome-Wide Association Study undertaken using a Mixed Linear Model approach on the average early root growth rate data from lines/cultivars grown on water agar with salt concentration of 200 mmol/l; data generated using an ANOVA. Depressions in marker significance observed in the center of each chromosome are due to reduced marker density around the centromere of the physical map.

The QQ plots for the early root growth data (Supplementary Figure 9) showed that, for the 0 mmol/l data, the EIGENSTRAT approach had the lowest deviation from the expected null distribution, whilst for the two salt concentrations the MLM approach did. The plots of the control data and the 100 mmol/l data (Supplementary Figure 10) showed no significance. The only significant marker found was in the 200 mmol/l plot (Figure 5) at the distal end of chromosome 3HS at 13.63 Mb (JHI_Hv50k_2016_154888), which had a high negative effect on the growth rate of −0.229 mm/day. Around this marker there were a total of ten E3 ubiquitin-protein ligases (Table 2), as well as: an AP2-containing protein, a calcium-transporting ATPase, a Pentatricopeptide repeat-containing protein, a phenylalanine ammonia-lyase 2, and an S-type anion channel (SLAH2).

**TABLE 2:** The name of the candidate genes identified in relation to salinity tolerance in regards to early root growth, with the chromosome and position on the physical map listed.

Gene Name	Chr	Position	Annotation
HORVU3Hr1G005260.1	3HS	13.04	E3 ubiquitin-protein ligase SINA-like 2
HORVU3Hr1G005270.2	3HS	13.05	E3 ubiquitin-protein ligase SINA-like 10
HORVU3Hr1G005290.3	3HS	13.06	E3 ubiquitin-protein ligase
HORVU3Hr1G005320.2	3HS	13.10	E3 ubiquitin-protein ligase SINA-like 5
HORVU3Hr1G005330.2	3HS	13.11	Phenylalanine ammonia-lyase 2
HORVU3Hr1G005380.1	3HS	13.22	E3 ubiquitin-protein ligase SINA-like 10
HORVU3Hr1G005400.7	3HS	13.43	E3 ubiquitin-protein ligase SINA-like 4
HORVU3Hr1G005410.2	3HS	13.43	E3 ubiquitin-protein ligase
HORVU3Hr1G005430.1	3HS	13.44	E3 ubiquitin-protein ligase SINA-like 11
HORVU3Hr1G005450.2	3HS	13.45	E3 ubiquitin-protein ligase SINA-like 5
HORVU3Hr1G005470.1	3HS	13.51	E3 ubiquitin-protein ligase SINA-like 10
HORVU3Hr1G005540.1	3HS	13.64	S-type anion channel SLAH2
HORVU3Hr1G005560.1	3HS	13.79	Pentatricopeptide repeat-containing protein
HORVU3Hr1G005580.1	3HS	13.81	AP2-containing protein
HORVU3Hr1G005590.1	3HS	13.82	Calcium-transporting ATPase, putative

Of these, the E3 ubiquitin-protein ligases were reasonably common comprising of 0.21% of the 73,586 genes listed in BARLEX the barley genome explorer (Colmsee et al., 2015), at 152 genes genome-wide. However, this region accounts for 6.6% of all E3 ubiquitin-protein ligases, and a third of the SINA-like genes. The Pentatricopeptide repeat-containing proteins are very common accounting for 0.7% of the genome, at 515 genes genome-wide. The remaining genes—encoding for AP2 proteins, calcium-transporting ATPases, S-type anion channels, and phenylalanine ammonia-lyases—comprises of between 0.05–0.01% of genes, at 39, 21, 19, and 9 genes, respectively.

### Comparisons

No correlations were found between the dry matter produced, germination speed, and early root growth rate, when comparing the values of the fitted slopes and the values in high salt concentrations. Lines identified in individual experiments as potential salt tolerant lines were then assessed on how they performed in the other screens undertaken (Table 3) and discussed below.

**TABLE 3:** Ten lines were selected as they displayed salinity tolerance in one or more of the experiments performed in this study.

		Genotype									
	NaCI levels	Bere 49 A 27 Shetland	Bere 55C 33	Bere-118	Craigs Triumph (SSRPB)-135	Floye	HSX07-26	Lawina	Nepal 92 BN-1	Prize Prolific-196	Scotch Common-M08
Dried shoot biomass (g) in compost	0 mmol/kg	18.3	14.2	10.3	7.5	24.0	14.9	17.0	8.4	16.3	12.2
	50 mmol/kg	22.8	9.3	14.0	7.3	27.1	16.5	17.4	13.1	20.8	9.6
	100 mmol/kg	20.0	14.7	13.5	4.7	15.2	4.0	6.0	5.9	15.7	11.6
Time of germination (hours) in agar	0 mmol/l	40	26	39	30	48	29	24	29	38	38
	100 mmol/l	41	31	50	33	48	31	22	36	48	34
	200 mmol/l	43	43	54	42	54	36	33	36	72	48
Early Root Growth (m/day) in agar	0 mmol/l	5.7	18.6	8.0	10.1	10.3	7.0	18.4	10.2	16.5	13.1
	100 mmol/l	4.0	3.9	2.7	3.6	4.3	0.7	3.5	1.3	4.2	3.1
	200 mmol/l	2.3	1.3	1.4	1.4	2.8	0.1	2.3	0.6	1.1	1.5

*Shoot biomass accumulation compared the dried shoot biomass after 70 days growth in compost with varying NaCl levels.*

*Germination speed compared the time of germination when grown in Petri dishes containing agar with varying NaCl levels.*

*Early root growth compared the speed of root growth from the germinated seeds mentioned above for the first 7 days.*

The line Bere 49 A 27 Shetland was identified in both the shoot biomass and root growth rate assessments for good performance in high salt conditions. It also showed no significant change in germination speed with increased salinity, however, it was slow to germinate even with no salinity at 40 h germination. The other line identified with a fast root growth rate in saline conditions was Floye, but this line performed below average when looking at the biomass growth and germination speed.

Other lines identified in the shoot biomass assessment were Prize Prolific-196 and Bere-118, both of which perform below average in the germination test, with rates of 54–72 h, but performed reasonably well in the root growth assay, comparable to the best performing elite line. When comparing between the salt levels Prize Prolific-196 showed root growth similar to the fastest growing lines at the 100 mmol/l salt concentration.

Of the lines identified with fast germination rates in salt conditions—Lawina, HSX07-26, and Nepal 92 BN-1—none showed tolerance in all measurements. Lawina had a fast root growth in salt conditions, but still diminished biomass compared to the control, and Nepal 92 BN-1 showed limited reductions in shoot biomass with increased salt, but the remainder showed average or below average performance in the other measurements.

Other lines that were not singled out in any experiment, but performed well throughout the study—maintaining high germination speeds, early root growth rates, and levels of biomass in both salt conditions—included Scotch Common-M08, Craigs Triumph (SSRPB)-135, and Bere 55C 33.

## Discussion

The problem of salt toxicity is limited to localized regions, though affects a large proportion (6.5%) of land worldwide (FAO, 2015), and is becoming an increasing problem with the irrigation of land with brackish water (Ayars et al., 1993; Umali, 1993; Wei et al., 2018), the onset of dryland salinity due to climate change (Rengasamy, 2006; Tomaz et al., 2020), and deforestation in temperate zones (Sahagian, 2000; Barbieri et al., 2020). There is a need to increase production on these increasingly marginal land areas that are being degraded by increasing salinities, where there are already problems in preserving yields. One method of elevating yield on these lands would be to increase the tolerance of the crops to salinity through breeding (Munns et al., 2006; Wu, 2018). For this to be successful salinity tolerance genes need to be identified, and a viable source of these genes could be from landraces that grow in marginal soils that can contain elevated salinity. This study has assessed landrace lines, focusing on the Scottish Bere, for their ability to maintain biomass, germination, early root growth, and other indicators, in saline conditions. This allowed for the identification of differences between lines—which follow overarching differences between the Bere, other landrace lines, and elite cultivars—as well as genomic regions associated with the maintenance of biomass in saline conditions, along with a number of genes with putative functions associated with salinity tolerance. However, it should be noted that only two-row elite cultivars were used, as opposed to six-row barley, such as Bere, that has been shown to be generally more tolerant (Kumar et al., 2020).

### Effect on Growth

A screening of the landrace collection showed that there were no differences in the way the genotypes interacted with the different salinities. However, when this data was fitted to a linear model to see how the different weights, height, and tiller number changed with increasing salinity it was seen that there was a significant difference between the dry weight when comparing both sub-categories and individual lines/cultivars. This revealed that in the elite cultivars the reduction in biomass was twice that of landrace lines with increasing salinity, with a loss of 0.39% per mmol/kg, suggesting that the elite cultivars are less tolerant to saline compost. This is comparable to the effect of salinity on dry weight from Long et al. (2013) that showed an average (of 192 genotypes) decrease in shoot dry weight of 67% from 0–200 mM NaCl (equivalent to 0–200 mmol/kg) in a hydroponic system, or 0.34% per mM. In this study the most salinity tolerant elite genotype lost 48% over the same range, or 0.24% per mM, which is more than the average of the Bere lines at 0.17%. However, it is possible that there were decreased levels of salinity in the compost of this study due to leaching. A similar experiment using gravel with nutrient solution with increasing salinity from Rawson et al. (1988) showed similar levels of decrease, with the most tolerant barley line having a 38% average loss in saline conditions (averaged 175–250 mM), or 0.18% per mM (Munns et al., 1995). When these results are shown individually it can be seen that the spread of the landrace lines, both Bere and non-Bere, is large, with the elite cultivars all above average. From these it was possible to identify a number of Bere and non-Bere landraces that have no, or positive, changes in dry weight with increasing salinity, suggesting that they are very salinity tolerant, similar result to those found by Rajeswari et al. (2019) who tested different barley accessions. This positive change in dry weight could be due to the effect of salinity on the availability of nutrients (Grattan and Grieve, 1992), providing a nutrient profile to which the lines are more adapted to. It is also possible to find Bere and non-Bere landraces that have very large negative changes, equal to the elite cultivars most affected by salinity, showing that the salinity tolerance is not a uniform trait across all landraces.

### Effects on Germination and Early Root Growth

When the lines/cultivars are looked at individually in terms of change in radicle emergence time with increasing salinity, results suggested that there some landrace lines are more tolerant to increased salinity than the elite cultivars. It is also seen that there is a correlation to the change in radicle emergence time with increased salinity and the radicle emergence time in the control. This suggests that lines that are faster germinating in control conditions have greater increases in germination time with increasing salinity, becoming more equivalent to the lines that were slower to germinate in the control. This correlation allowed for identification of lines that were both quick germinating in the control conditions and had a reduced rate of increase in radicle emergence time with increased salinity: Lawina, HSX07-26, Nepal 92 BN-1, and Tiree six row 12 A. This suggests that these lines have an increased resistance, at the germination stage, to elevated salinity without a reduction in germination speed that would be detrimental. Whilst it has been shown that the genes involved in resistance at the germination stage are different from those at the seedling and later stages (Mano and Takeda, 1997), it can be seen in a screen of Algerian landraces that the lines that were identified as salinity tolerant at seedling growth also had high germination speeds in saline conditions, though not all with high germination speeds showed salinity tolerance at seedling growth (Adjel et al., 2013). However, few studies have identified barley QTLs linked to salinity tolerance during germination (Thabet et al., 2021).

Landrace screen results for the early root growth show clear differences between the sub-categories, with the Bere lines on average growing quicker and larger with all salinities and the elite cultivars showing the opposite. This suggests that the Bere lines have, on average, quicker growing roots that are not as affected by salinity as the elite and other landraces. Individual lines/cultivars showed significant differences between the lines/cultivars and how they reacted to saline conditions, but it was shown that there was no correlation between root growth rates in any of the media. However, it was possible to identify lines that showed high rates of root growth in both medium and high salinities, some due to having high levels of root growth in control conditions, such as Floye, and others despite not, such as Bere 49 A 27 Shetland. The latter of these are of most interesting as it has been shown that the maintenance of root elongation, in saline conditions that inhibit the normal shoot growth of the seedling, is indicative of an adaptive method to safeguard uptake of nutrients and water (Shelden et al., 2020), with other experiments showing salinity tolerant lines having shorter roots in control conditions but maintaining them in saline conditions (Adjel et al., 2013; Nefissi Ouertani et al., 2021).

### Genome Associations With Salinity Tolerance

The GWAS undertaken on the shoot biomass identified one significant QTL of interest at 5HL. Within the region at 5HL there are a number of genes encoding for proteins of interest such as a) Lysine-specific demethylase REF6—selected as it has a histone demethylase domain and over expression of a histone demethylase gene in *Arabidopsis* has been shown to improve salinity tolerance (Shen et al., 2014) as well as associations of a histone demethylase family with salinity tolerance in cotton (Sun et al., 2021); b) Actin 7—selected as salinity stress has been shown to affect actin filament assembly and has shown to be necessary in salinity tolerance in *Arabidopsis* (Wang et al., 2011); c) Ferredoxin 3—selected as an overexpression of plant ferredoxin-like protein has been seen to promote salinity tolerance in rice (Huang et al., 2020), and salinity stress has been associated with ferredoxin associated proteins (Berteli et al., 1995; Zhou et al., 2009); and d) Acyl-CoA-binding domain-containing protein 4—selected as Acyl-CoA-binding proteins have been shown to interact with other proteins in response to abiotic stresses (Raboanatahiry et al., 2015), with overexpression in *Arabidopsis* shown to improve drought tolerance (Du et al., 2016) and expression of maize Acyl-CoA-binding proteins in *Arabidopsis* increasing resistance to salinity and drought stress (Zhu et al., 2021).

The GWAS performed on the early root growth in each of the salinities, compared with the control to identify the peaks that are connected with root growth in general, reveal only one significant marker positioned on chromosome 3HS. Xu et al. (2012) also found minor QTLs on 3H, but not in the same position. Around this marker there were ten genes encoding for E3 ubiquitin-protein ligases, selected due to both the positive and negative association with salinity stress, drought stress, or general abiotic stress in *Arabidopsis* in multiple different ways (Qin et al., 2008; Seo et al., 2012; Li et al., 2013; Cho et al., 2017; Zhang et al., 2017; Liu et al., 2020). Other candidates include genes encoding for: (a) AP2-containing protein—as AP2-containing proteins have been found to be expressed in *Arabidopsis* root tissue during high salinity stress (Sakuma et al., 2002) and found to be released under salinity and drought stress and bind to Dehydration-Responsive Element that regulate stress response in rice (Tian et al., 2005; Todaka et al., 2015); (b) calcium-transporting ATPase—as these have been shown to be key to adaptation of plants to biotic and abiotic stresses including salinity stress (Kabała and Kłobus, 2005; Bose et al., 2011) due to the regulation of calcium signaling (Kumar et al., 2021). *Arabidopsis* calcium-transporting ATPase have been shown to convey salinity resistance to yeast when transferred (Anil et al., 2008). It has also been highlighted as a candidate gene for salinity tolerance in wheat landraces (Borjigin et al., 2021); (c) Pentatricopeptide repeat (PPR)-containing protein—as PPR containing proteins have been shown to be involved in the response to biotic and abiotic stresses in *Arabidopsis* (Laluk et al., 2011), specifically improving salinity tolerance in *Arabidopsis* (Zsigmond et al., 2012), as well as in other plants (Hajrah et al., 2017; Xing et al., 2018); (d) phenylalanine ammonia-lyase (PAL) 2—as PAL activity has been influenced by abiotic and biotic stress, including salinity stress, in a number of different plants (MacDonald and D’Cunha, 2007; Gao et al., 2008; Dehghan et al., 2014; Nag and Kumaria, 2018) including maize (Gholizadeh and Kohnehrouz, 2010), though a study by Cass et al. (2015) has also shown no effect on the related drought stress when knocked-out in the temperate cereal crop model *Brachypodium distachyon*; and (e) S-type anion channel SLAH2—as SLAH1 have been shown to regulate Cl^–^ accumulation under salinity stress in *Arabidopsis* (Roy et al., 2016) and confer salinity tolerance by shoot Cl^–^ accumulation (Cubero-Font et al., 2016; Colmenero-Flores et al., 2019).

HKT genes are the most recognized genes for salinity tolerance (Hamamoto et al., 2015; Hazzouri et al., 2018), with associated salinity tolerance in barley (Mian et al., 2011; Han et al., 2018; Van Bezouw et al., 2019), possibly due to negative regulation (Huang et al., 2019). However, none were identified in the QTL regions identified in this study, indicating different mechanisms like those listed above through either tissue tolerance or different methods of exclusion (Munns, 2009).

### Potential of Landraces as a Source of Salinity Tolerance

Further testing of the identified lines should be undertaken to gain a greater understanding into the nature and extent of the salinity tolerance. This could include assessing the root growth in compost or soil of differing salinities, assessing the Na concentrations within the leaves grown in these concentrations, and assessing the yield when grown in these concentrations. Full cropping lifecycle assessments will be crucial in understanding the importance of individual traits tested here to salinity tolerant crops. The lines identified, particularly Bere 49 A 27 Shetland, could be used directly for soils that are highly salinity affected such as coastal soils and areas irrigated with poor quality water. Additionally, further testing on these lines with ratios of differing salts is necessary as whilst sodium salts are the most common other salts are also found in soils. These salts are predominantly chlorides and sulfates of minerals such as calcium, potassium, and magnesium, but also include carbonates, bicarbonates, and nitrates of these minerals (Abrol et al., 1988; Chaurasia et al., 2018). This is particularly necessary as the ratio of sodium salt to other salts is much lower in costal saline soils that are sea influenced (Mugai, 2004), such as some Scottish islands where Bere barley is grown (Dry and Robertson, 1982; Dry, 2016).

Once a more detailed understanding of the salinity tolerance has been attained, the regions that have been identified in this study can then be introgressed into elite cultivars. Introgressed lines would help further isolate the gene(s) associated with the identified resistance, which could then be bred into an elite background. These lines would be able to alleviate both salinity and drought stress. They would be of particular importance in geographical regions without adequate water supplies as the most common reason for the necessity of watering with brackish water is the prevalence of drought (Hillel, 2000; Ismail and Horie, 2017; Wei et al., 2022), and will further allow for the use of saline waters to alleviate drought stress from source such as the Mediterranean (Hamdy et al., 2005; Munns et al., 2020). This is of increased importance as areas of salinity and drought are expected to increase over the upcoming years (Wang et al., 2003), exacerbated by climate change causing unpredictable weather and rising water tables (Munns and Gilliham, 2015; Corwin, 2021). Salinity stress is also highly related to drought stress, both having similar or identical effect on water deficiency and osmotic effect (Hu and Schmidhalter, 2005; Katerji et al., 2009; Manna et al., 2021), thus the identified mechanisms that offer an increase in salinity tolerance should be assessed for drought stress tolerance, particularly those lines found in the germination trials.

## Conclusion

This study set out to show the potential of landraces in adapting to high salinities, with a focus on European landraces and Scottish Bere, which had no prior salinity tolerance associated (Kumar et al., 2020), compared to the European elite cultivars used that showed less tolerance than most of the landraces used.

Differences in the type of salinity stress between experiments could explain the how some lines identified as salinity tolerant in one experiment were not identified in others. Evidence of this has been found by Rahnama et al. (2011) and Shelden et al. (2013) who showed that salinity effected root growth rate at the seedling stage, but this did not correlate with the ion concentration in the shoot and root tissue, indicating that the tolerance is to osmotic stress. By combining different mechanisms used to tolerate the different types of salinity stress it is possible to improve the salinity tolerance of a crop further (Roy et al., 2014). The different lines and regions identified in this study can be further studied to help identify different mechanisms to salinity tolerance, and could then be used to build a more robust resistance by combining multiple mechanisms within a crop. Bere 49 A 27 Shetland showed salinity tolerance in all experiment (and Bere 55C 33 with lower salinity stress) and thus shows strong potential for finding different tolerance mechanisms.

These tolerance mechanisms to salinity will be crucial in future years due to the increase in salinity expected due to the continued effects of man-made climate change; such as rising salinity in the ground water due to sea level rises, and increase use of brackish water for irrigation due to drought caused by shifting weather patterns.

## Data Availability Statement

The original contributions presented in this study are included in the article/Supplementary Material, further inquiries can be directed to the corresponding author/s.

## Author Contributions

JC contributed to the experimental work, analysis, and manuscript writing. GN, TG, and AN provided supervision and advice throughout the project, as well as significant editorial decisions in creating the manuscript. All authors contributed to the article and approved the submitted version.

## Conflict of Interest

The authors declare that the research was conducted in the absence of any commercial or financial relationships that could be construed as a potential conflict of interest.

## Publisher’s Note

All claims expressed in this article are solely those of the authors and do not necessarily represent those of their affiliated organizations, or those of the publisher, the editors and the reviewers. Any product that may be evaluated in this article, or claim that may be made by its manufacturer, is not guaranteed or endorsed by the publisher.

## Supplementary Material

Supplementary Figure 1 Quantile-quantile (QQ) plots of the expected vs the observed degrees of freedom for the Genome-Wide Association Study undertaken using a Mixed Linear Model approach **(A,C)** or EIGENSTRAT **(B,D)**; for the datasets of average slope fitted to saline compost raw data **(A,B)**, and as a percentage of the control **(C,D)**. The expected null distribution is represented by the black line, with a line of best fit for the plotted data represented in red.

Supplementary Figure 2 Average time for radicle to emerge in Petri dishes 140 lines/cultivars of barley divided into three, unequal, sub-divisions (36, 99, and 5 for the Bere, other landraces, and elites, respectively) over three differing salt concentrations. Error bars represent the standard errors in positive and negative directions.

Supplementary Figure 3 Average early root length in Petri dishes for 140 lines/cultivars of barley divided into three, unequal, sub-divisions (36, 99, and 5 for the Bere, other landraces, and elites, respectively) over three differing salt concentrations. Error bars represent the standard errors in positive and negative directions.

Supplementary Figure 4 Early root growth rate of lines/cultivars grown water agar with salt concentration of 200 mmol/l. Lines identified are referred to in the text. Error bars represent the standard errors in positive and negative directions.

Supplementary Figure 5 Quantile-quantile (QQ) plots of the expected vs the observed degrees of freedom for the Genome-Wide Association Study undertaken using a Mixed Linear Model approach **(A,C,E)** or EIGENSTRAT **(B,D,F)**; for the datasets of average radicle emergence time for samples grown in 0 **(A,B)**, 100 **(C,D)**, and 200 mmol/l **(E,F)**. The expected null distribution is represented by the black line, with a line of best fit for the plotted data represented in red.

Supplementary Figure 6 A Manhattan plot of a Genome-Wide Association Study undertaken using an EIGENSTRAT approach on the average time for radicle emergence data from lines/cultivars grown on water agar with salt concentration of **(A)** 0, **(B)** 100, and **(C)** 200 mmol/l; data generated using an ANOVA. Depressions in marker significance observed in the center of each chromosome are due to reduced marker density around the centromere of the physical map.

Supplementary Figure 7 A Manhattan plot of a Genome-Wide Association Study undertaken using a Mixed Linear Model approach on the average increase in radicle emergence time with increased salt concentration; data generated using an ANOVA. Depressions in marker significance observed in the center of each chromosome are due to reduced marker density around the centromere of the physical map.

Supplementary Figure 8 Quantile-quantile (QQ) plots of the expected vs the observed degrees of freedom for the Genome-Wide Association Study undertaken using a Mixed Linear Model approach **(A)** or EIGENSTRAT **(B)**; for the datasets of average increase in radicle emergence time with increase salt concentrations. The expected null distribution is represented by the black line, with a line of best fit for the plotted data represented in red.

Supplementary Figure 9 Quantile-quantile (QQ) plots of the expected vs the observed degrees of freedom for the Genome-Wide Association Study undertaken using a Mixed Linear Model approach **(A,C,E)** or EIGENSTRAT **(B,D,F)**; for the datasets of average early root growth rate for samples grown in 0 **(A,B)**, 100 **(C,D)**, and 200 mmol/l **(E,F)**. The expected null distribution is represented by the black line, with a line of best fit for the plotted data represented in red.

Supplementary Figure 10 A Manhattan plot of a Genome-Wide Association Study undertaken using an EIGENSTRAT **(A)** or Mixed Linear Model **(B)** approach on the average early root growth rate data from lines/cultivars grown on water agar with salt concentration of **(A)** 0, and **(B)** 100 mmol/l; data generated using an ANOVA. Depressions in marker significance observed in the center of each chromosome are due to reduced marker density around the centromere of the physical map.

## Abbreviations

ANOVA: Analysis of variance
ATP: Adenosine triphosphate
FAO: Food and Agriculture Organization of the United Nations
GWAS: Genome-wide association study
HKT: High-affinity Potassium
IBS: Identical by state
JHI: James Hutton Institute
JHI-SBLC: JHI Heritage Spring Barley Landrace Collection
JIC-GRU: John Innes Centre Germplasm Resources Unit
LOD: Logarithm of odds
PAL: Phenylalanine ammonia-lyase
PPR: Pentatricopeptide repeat
QQ: Quantile-quantile
QTL: Quantitative trait loci
SASA: Science and Advice for Scottish Agriculture
SNP: Single-nucleotide polymorphism.
