# Supplementary material for: Evaluating Variation in Germination and Growth of Landraces of Barley (Hordeum vulgare L.) Under Salinity Stress
Source: Front Plant Sci. 2022 Jun 16;13:863069. doi: 10.3389/fpls.2022.863069 (PMC9245355; doi:10.3389/fpls.2022.863069)
Supplement: Supplementary Figure 1 — Quantile-quantile (QQ) plots of the expected vs the observed degrees of freedom for the Genome-Wide Association Study undertaken using a Mixed Linear Model approach (A,C) or EIGENSTRAT (B,D); for the datasets of average slope fitted to saline compost raw data (A,B), and as a percentage of the control (C,D). The expected null distribution is represented by the black line, with a line of best fit for the plotted data represented in red. [file Data_Sheet_1.PDF]

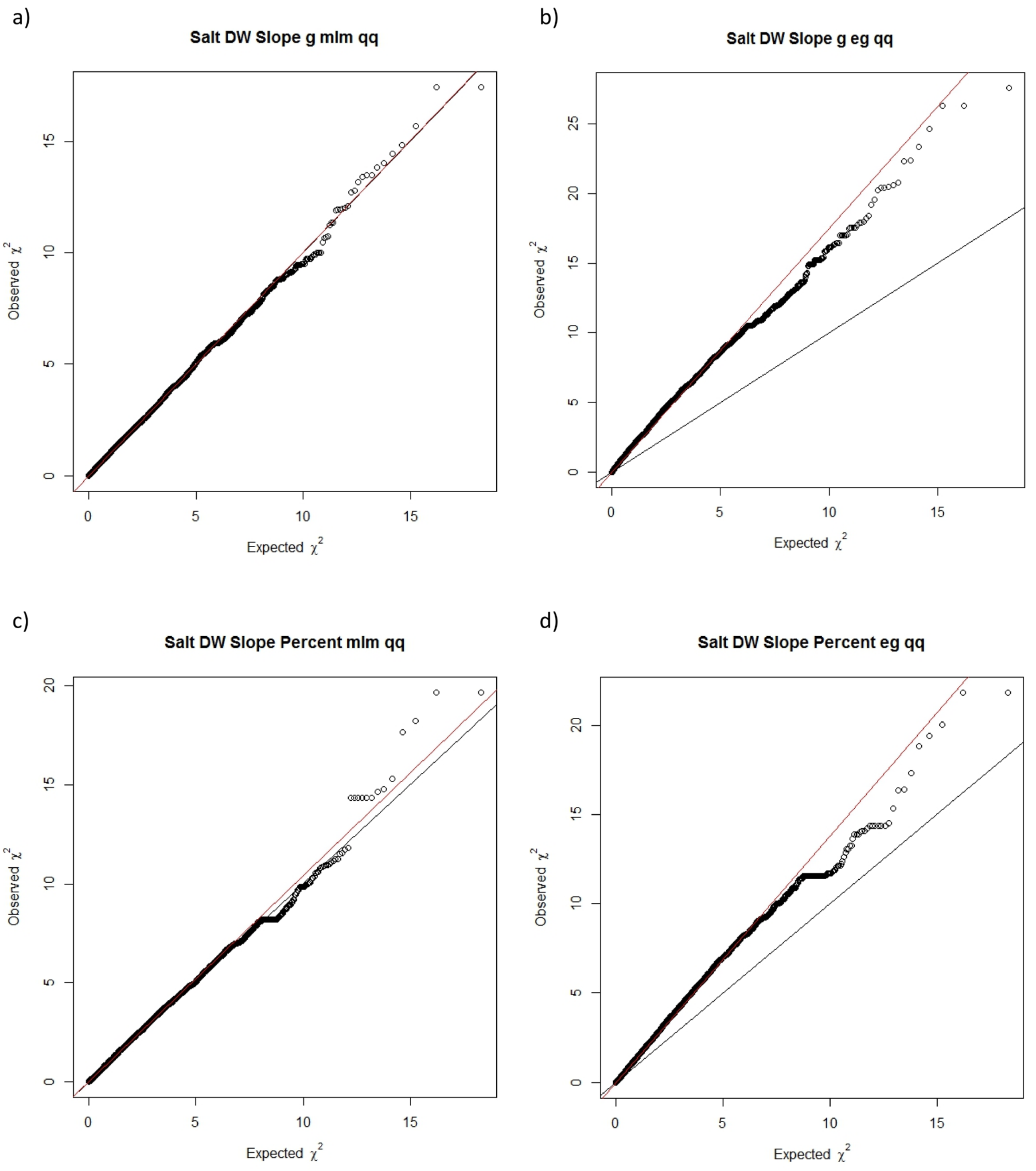

Supplementary Figure 1

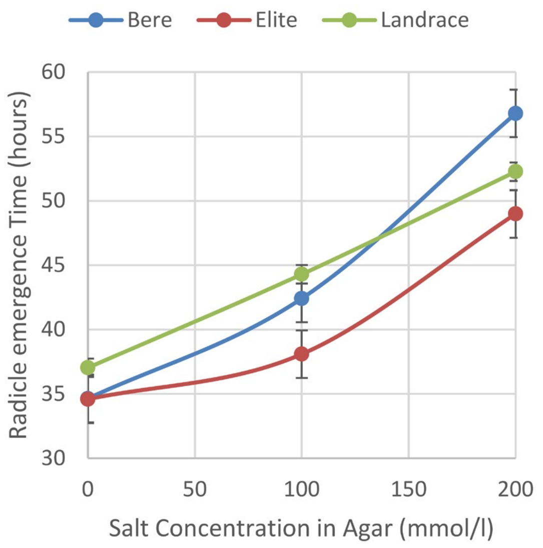

Supplementary Figure 2

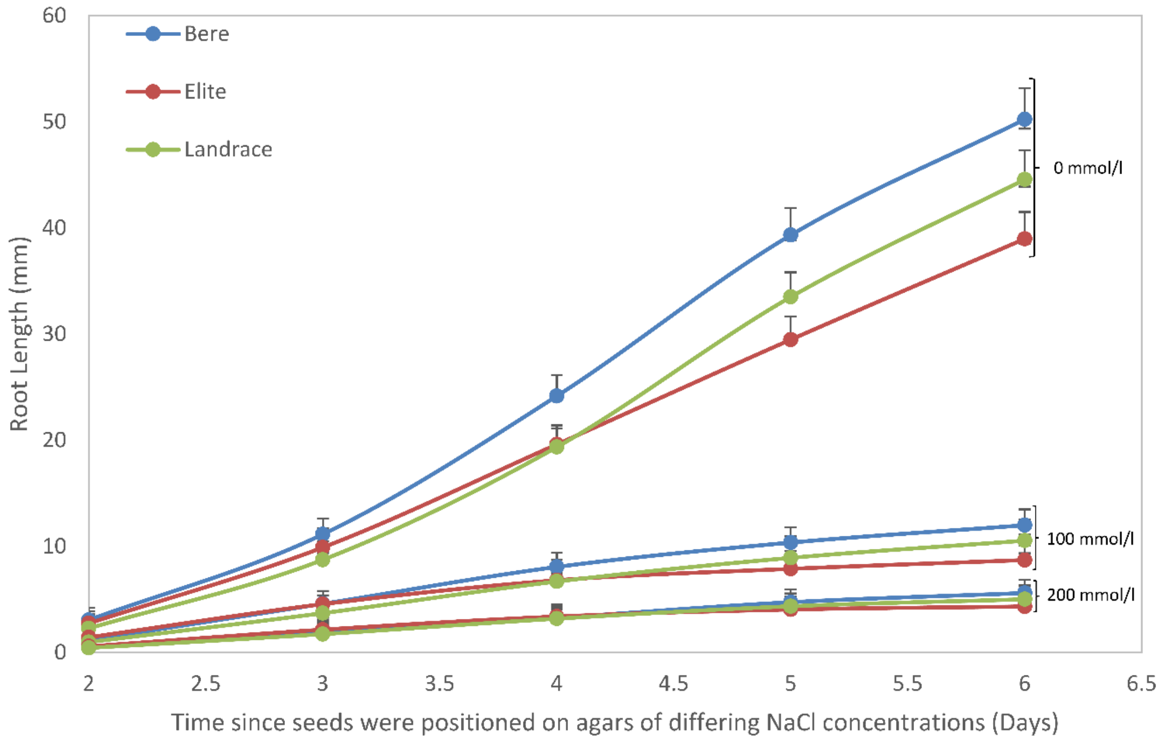

Supplementary Figure 3

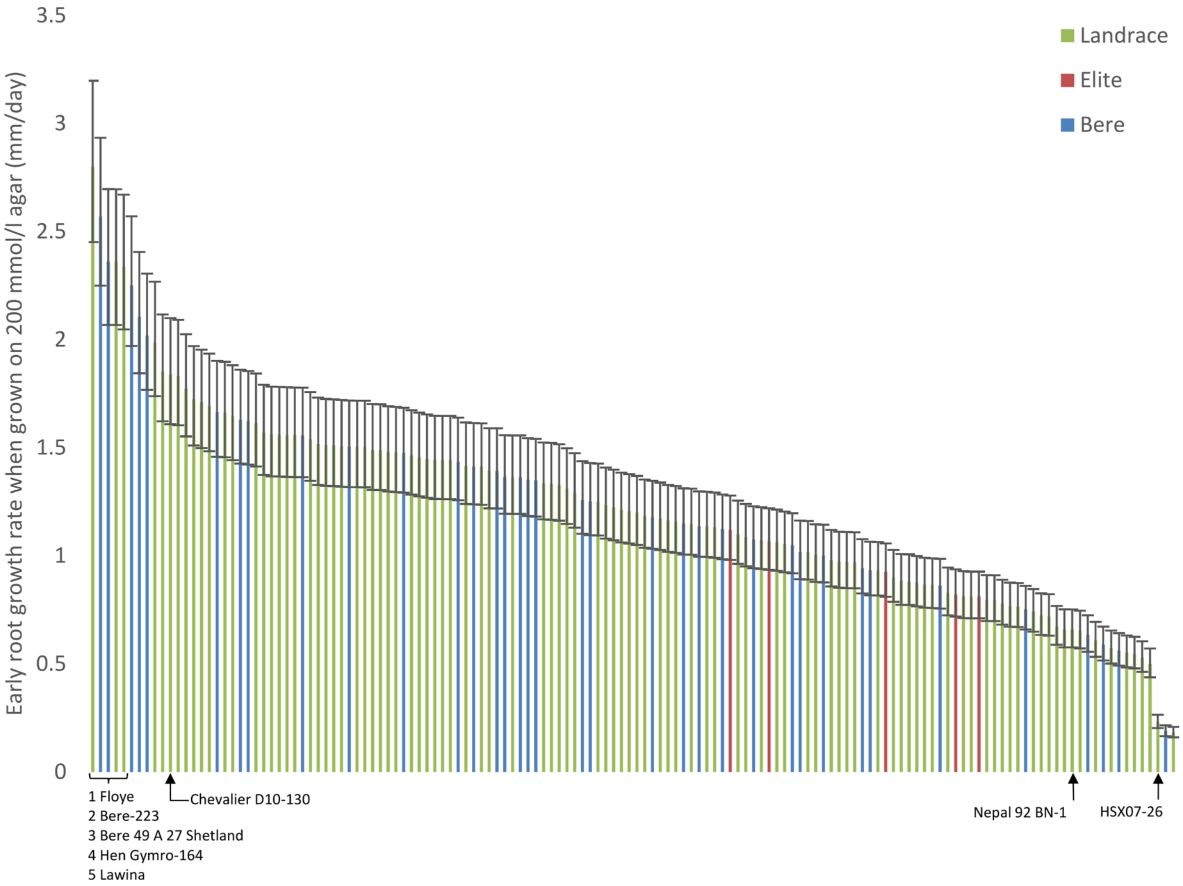

Supplementary Figure 4

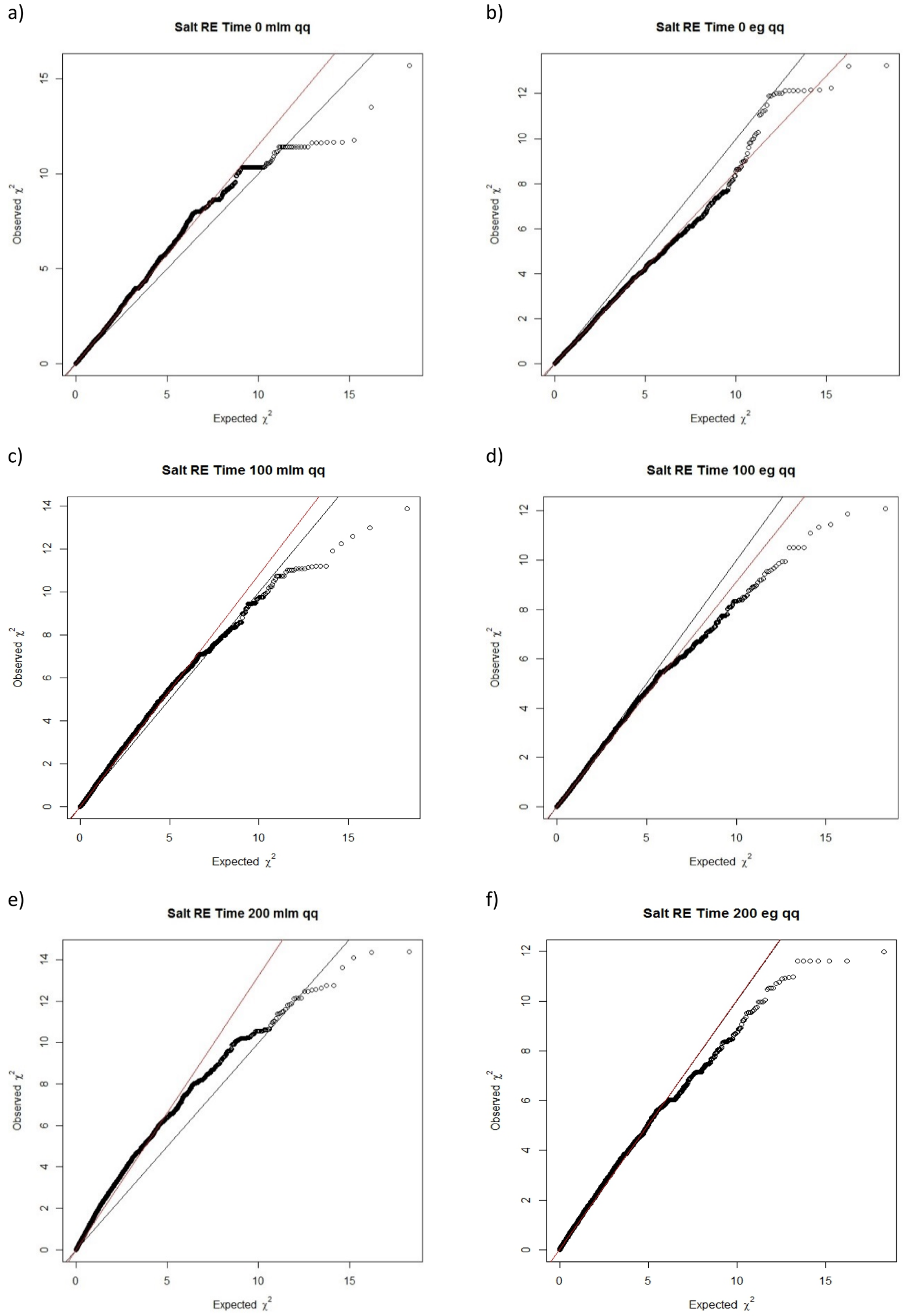

Supplementary Figure 5

a)

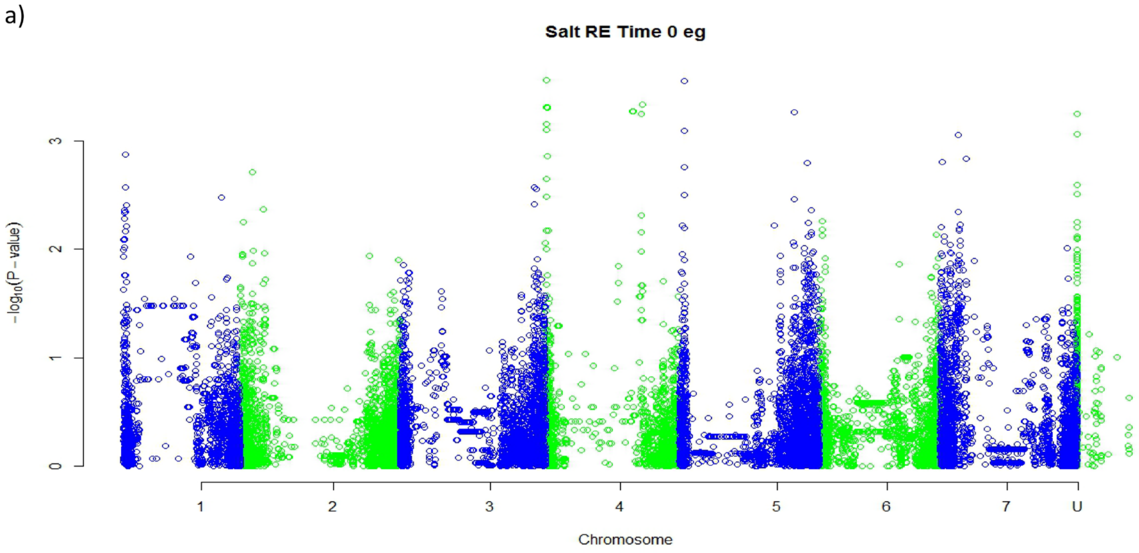

b)

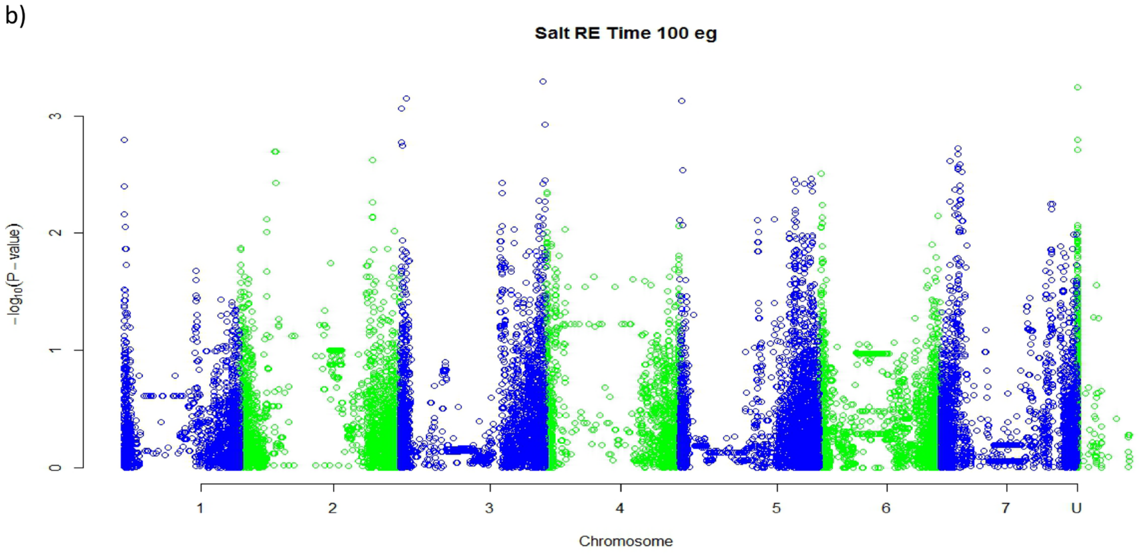

c)

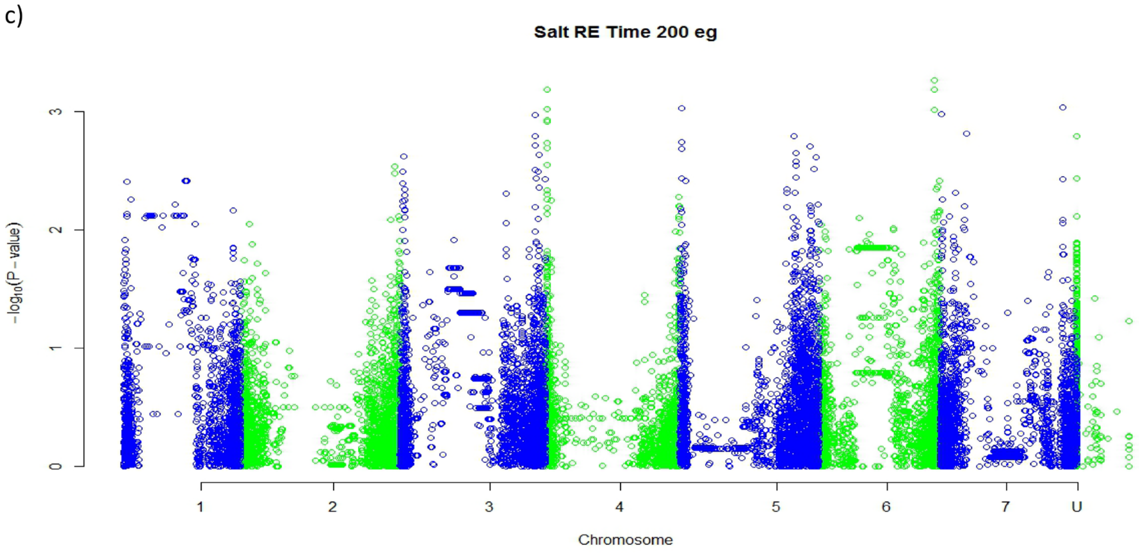

Supplementary Figure 6

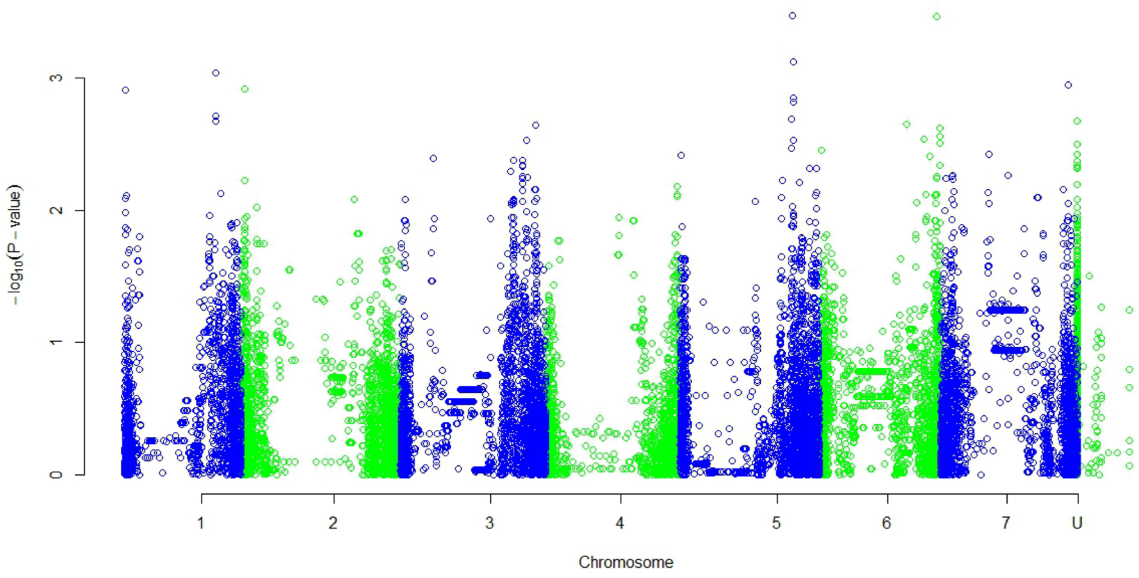

Supplementary Figure 7

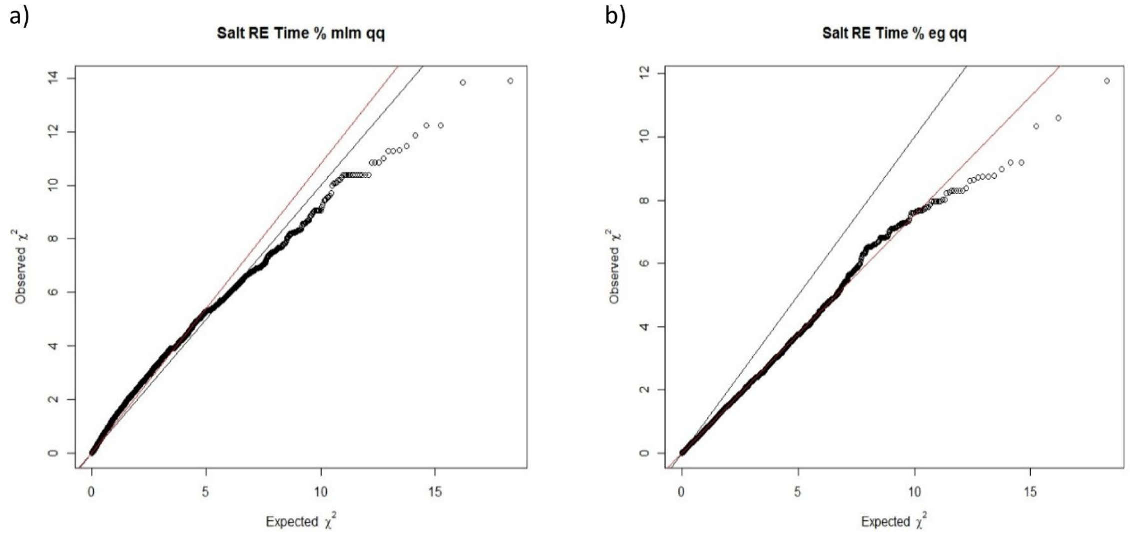

Supplementary Figure 8

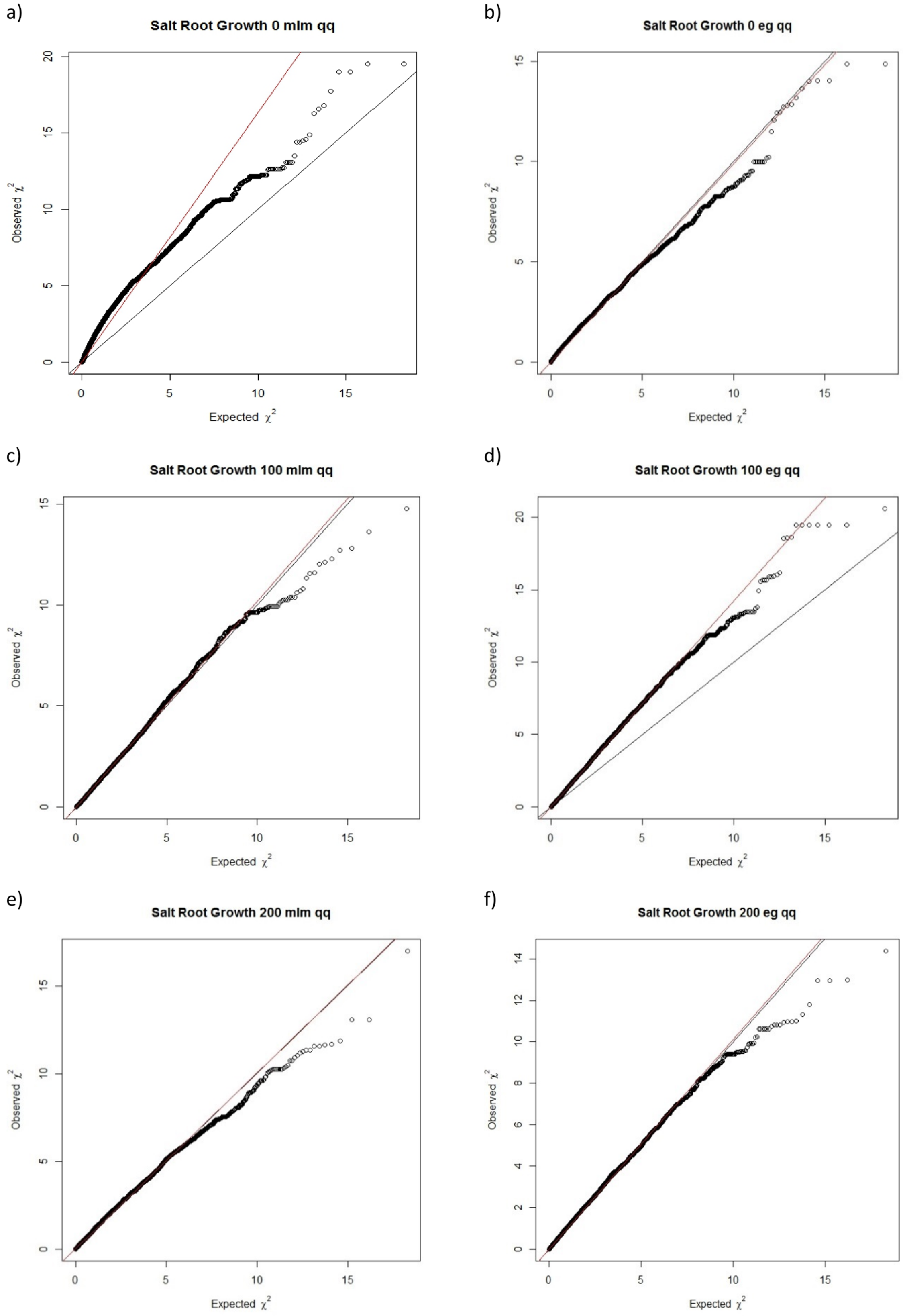

Supplementary Figure 9

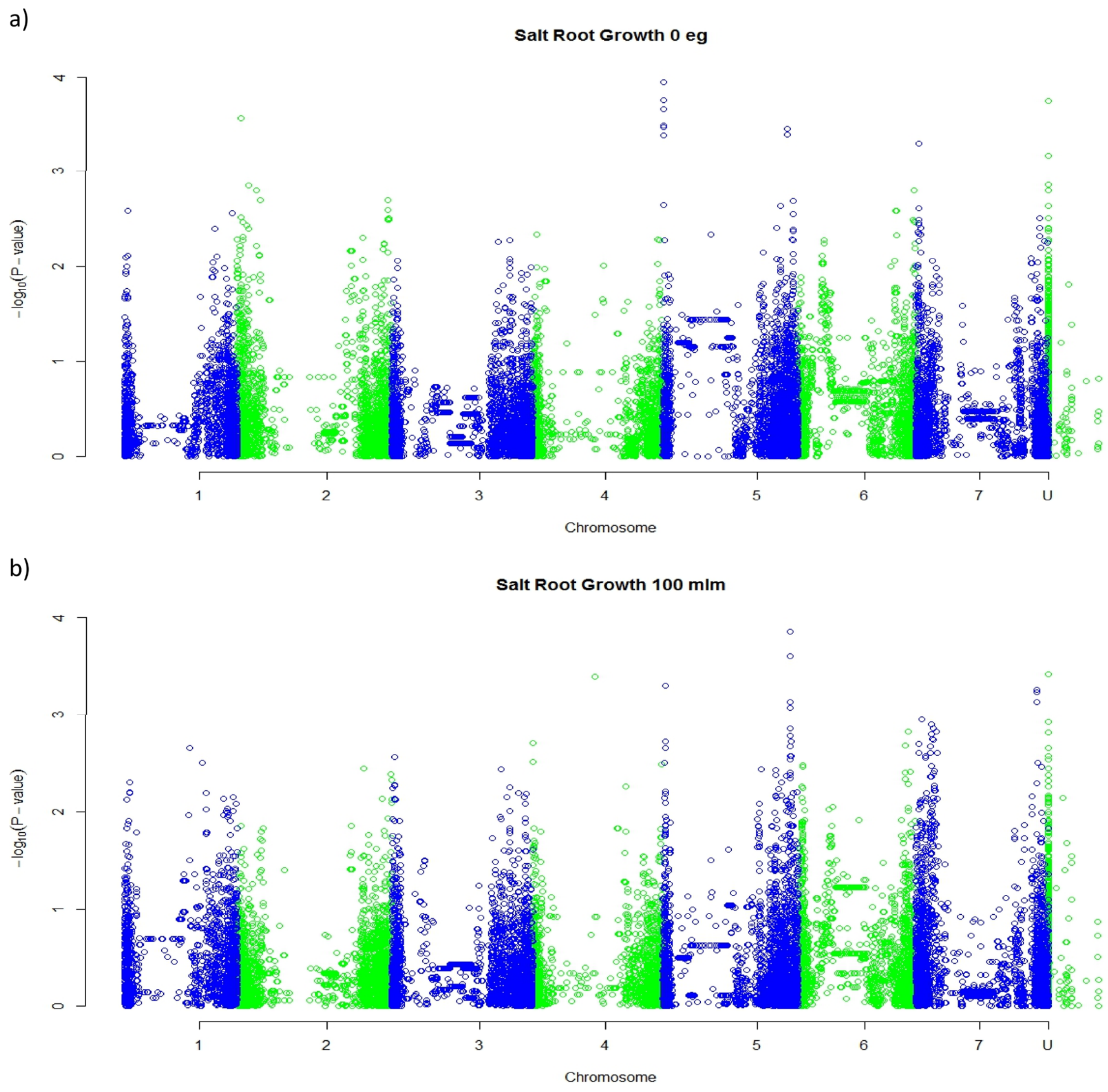

Supplementary Figure 10
