# Supplementary material for: Evaluating Variation in Germination and Growth of Landraces of Barley (Hordeum vulgare L.) Under Salinity Stress
Source: Front Plant Sci. 2022 Jun 16;13:863069. doi: 10.3389/fpls.2022.863069 (PMC9245355; doi:10.3389/fpls.2022.863069)
Supplement: Supplementary file 2 [file Data_Sheet_2.docx]

Supplementary Table 1) A subset of 140 lines from the James Hutton Spring Barley Collection that were chosen to be screened for salinity resistance in both the Shoot Biomass Growth trials, and the Germination & Early Root Growth Trials (the lines highlighted in bold were not used in the latter).

| Afghan 1169 | Bere-116 | Goldfield-151 | **Old Wiltshire-186** |
| --- | --- | --- | --- |
| Aramir-M08 | Bere-118 | Goldfield-152 | **Opal-188** |
| Aurore-107 | Bere-119 | Goldthorpe-153 | Optic |
| Balder-108 | Bere-120 | Gull-158 | Pembroke-190 |
| Beavans 35/51-110 | Bere-155 | Haidd Garw-159 | Plumage Archer Selection-M08 |
| Beavans 35-109 | Bere-223 | Hanna-M08 | Plumage-192 |
| Bere (Mr SO)-121 | Bere-M08 | Hen Gymro-161 | Plumage-M08 |
| Bere (Scots)-122 | Binder-M08 | Hen Gymro-164 | Prior-195 |
| Bere 23 A | Bonus-127 | Hen Gymro-165 | Prize Prolific-196 |
| Bere 23 B | Bowman | Hen Haidd Enlli | Proctor-M08 |
| Bere 23 C | Burtons Malting-128 | Hen Hardd Eulii 78 A | Rene Guillemart-197 |
| Bere 24268 A 71 | BW 902 | Hen Hardd Eulii 78 C | Rigel-199 |
| Bere 25 A | Camton-129 | Hindukusch | SASA 27 A Bere North Uist |
| Bere 2962 (AB) | Carslberg-M08 | HSX07-15 | Scotch Common-M08 |
| Bere 37 A 14 | Chevalier D10-130 | HSX07-20 | Scottish Annat-202 |
| Bere 39 A 16 Berneray | Chevalier-M08 | HSX07-26 | Scottish Common 7083 |
| Bere 43 A 21 | China Huang Yen | Irish Goldthorpe-171 | Scottish Common 7683 |
| Bere 44 A 22 | Common-132 | Irish Goldthorpe-172 | Skadu Local ''Oldings'' |
| Bere 45 A 23 | Common-218 | Irish Goldthorpe-222 | Spratt Archer 37/6/3-205 |
| Bere 47 A 25 | Cornish-133 | Isaria | Spratt Archer-M08 |
| Bere 4828 A 63 | Craigs Triumph (SSRPB)-135 | Japan Kitagawa Chobo | Spratt-M08 |
| Bere 49 A 27 Shetland | Danubia | Kenia-M08 | St Davids-206 |
| Bere 52 A 30 | Earl-140 | Laevigatum-175 | Stat -Old 14 |
| Bere 53 A 31 | Early Welsh-141 | Lawina | Swanneck-210 |
| Bere 55 A 33 | Eire Six Row-143 | Lenta-176 | Swannek-212 |
| Bere 55C 33 | Eire Six Row-220 | Long Eared Nottingham-177 | Swannek-213 |
| Bere 58 A 36 Eday | Floye | **Maja-179** | Tibet37 |
| Bere 59 A 37 Uist | Gartons Archer-144 | Morayshire Gold 7009 | Tiree six row 12 (AB) |
| Bere 60 A | Glasnevin 1-145 | Morex | Tiree six row 12 A |
| Bere 7045 (AB) | Gold-146 | Nepal 92 BN-1 | Vollkorngerste-214 |
| Bere A 3962 62 | Golden Archer-147 | NFC Tipple | Webbs Binder-215 |
| Bere-112 | Golden Drop-148 | Northumberland Rogue-182 | Webbs Burton Malting-216 |
| **Bere-113** | Golden Melon-149 | Old Cromarty-183 | Webbs Naked 2-Row-217 |
| Bere-114 | Golden Pheasant-150 | Old Irish-184 | Westminster |
| Bere-115 | **Golden Promise-M08** | Old Irish-221 | Zephyr-M08 |

Supplementary Table 2) Salt screen comparing changes in dry biomass weight over increasing NaCl concentrations, with line/cultivar sub-groups (Bere, other landraces, and elites; denoted as “Type”) as the variate. Using the replicate (denoted as “Exp”) as the blocking factor.

Change d.f. s.s. m.s. v.r. F pr.

+ Exp 7 0.667738 0.095391 22.80 <.001

+ Type 2 0.030153 0.015077 3.60 0.028

Residual 548 2.292300 0.004183

Total 557 2.990191 0.005368

Supplementary Table 3) Salt screen comparing changes in tiller number over increasing NaCl concentrations, with line/cultivar sub-groups (Bere, other landraces, and elites; denoted as “Type”) as the variate. Using the replicate (denoted as “Exp”) as the blocking factor.

Change d.f. s.s. m.s. v.r. F pr.

+ Exp 7 0.275056 0.039294 8.44 <.001

+ Type 2 0.024824 0.012412 2.67 0.070

Residual 548 2.552102 0.004657

Total 557 2.851982 0.005120

Supplementary Table 4) Salt screen comparing changes in fresh biomass weight over increasing NaCl concentrations, with line/cultivar sub-groups (Bere, other landraces, and elites; denoted as “Type”) as the variate. Using the replicate (denoted as “Exp”) as the blocking factor.

Change d.f. s.s. m.s. v.r. F pr.

+ Exp 7 10.3665 1.4809 7.23 <.001

+ Type 2 0.5887 0.2943 1.44 0.239

Residual 548 112.2799 0.2049

Total 557 123.2351 0.2212

Supplementary Table 5) Salt screen comparing changes in height over increasing NaCl concentrations, with line/cultivar sub-groups (Bere, other landraces, and elites; denoted as “Type”) as the variate. Using the replicate (denoted as “Exp”) as the blocking factor.

Change d.f. s.s. m.s. v.r. F pr.

+ Exp 7 2.62763 0.37538 7.77 <.001

+ Type 2 0.19984 0.09992 2.07 0.127

Residual 548 26.46530 0.04829

Total 557 29.29278 0.05259

Supplementary Table 6) Salt screen comparing changes in dry biomass weight over increasing NaCl concentrations, with line/cultivar (denoted as “Genotype”) as the variate. Using the replicate (denoted as “Exp”) as the blocking factor.

Change d.f. s.s. m.s. v.r. F pr.

+ Exp 7 0.667738 0.095391 25.76 <.001

+ Genotype 144 0.819052 0.005688 1.54 <.001

Residual 406 1.503401 0.003703

Total 557 2.990191 0.005368

Supplementary Table 7) Salt screen comparing changes in fresh biomass weight over increasing NaCl concentrations, with line/cultivar (denoted as “Genotype”) as the variate. Using the replicate (denoted as “Exp”) as the blocking factor.

Change d.f. s.s. m.s. v.r. F pr.

+ Exp 7 10.3665 1.4809 7.89 <.001

+ Genotype 144 36.6296 0.2544 1.35 0.011

Residual 406 76.2390 0.1878

Total 557 123.2351 0.2212

Supplementary Table 8) Salt screen comparing changes in dry biomass as a percentage of the control over increasing NaCl concentrations, with line/cultivar (denoted as “Genotype”) as the variate. Using the replicate (denoted as “Exp”) as the blocking factor.

Change d.f. s.s. m.s. v.r. F pr.

+ Exp 7 26.4911 3.7844 13.50 <.001

+ Genotype 144 51.0019 0.3542 1.26 0.040

Residual 404 113.2260 0.2803

Total 555 190.7191 0.3436

Supplementary Table 9) Salt screen comparing changes in fresh biomass as a percentage of the control over increasing NaCl concentrations, with line/cultivar (denoted as “Genotype”) as the variate. Using the replicate (denoted as “Exp”) as the blocking factor.

Change d.f. s.s. m.s. v.r. F pr.

+ Exp 7 20.1423 2.8775 8.09 <.001

+ Genotype 144 69.9013 0.4854 1.36 0.010

Residual 404 143.7464 0.3558

Total 555 233.7900 0.4212

Supplementary Table 10) Salt germination screen comparing radicle emergence times, with line/cultivar sub-groups (Bere, other landraces, and elites; denoted as “Type”) and NaCl concentration (denoted as “Conc”) as the variates. Using the replicate (denoted as “Rep stratum”) as the blocking factor.

Source of variation d.f. (m.v.) s.s. m.s. v.r. F pr.

Rep stratum 5 9510.8 1902.2 6.20

Rep.*Units* stratum

Type 2 3887.0 1943.5 6.33 0.002

Conc 2 128256.9 64128.4 208.95 <.001

Type.Conc 4 5334.0 1333.5 4.34 0.002

Residual 2170 (516) 666003.0 306.9

Total 2183 (516) 781392.0

Supplementary Table 11) Salt germination screen comparing coleoptile emergence times, with line/cultivar sub-groups (Bere, other landraces, and elites; denoted as “Type”) and NaCl concentration (denoted as “Conc”) as the variates. Using the replicate (denoted as “Rep stratum”) as the blocking factor.

Source of variation d.f. (m.v.) s.s. m.s. v.r. F pr.

Rep stratum 5 62665.9 12533.2 44.03

Rep.*Units* stratum

Type 2 3024.9 1512.5 5.31 0.005

Conc 2 735599.0 367799.5 1292.19 <.001

Type.Conc 4 2179.2 544.8 1.91 0.106

Residual 1705 (981) 485298.0 284.6

Total 1718 (981) 942140.8

Supplementary Table 12) Salt germination screen comparing radicle emergence times, with line/cultivar (denoted as “Genotype”) and NaCl concentration (denoted as “Conc”) as the variates. Using the replicate (denoted as “Rep stratum”) as the blocking factor.

Source of variation d.f. (m.v.) s.s. m.s. v.r. F pr.

Rep stratum 5 9242.2 1848.4 7.36

Rep.*Units* stratum

Genotype 139 200239.4 1440.6 5.73 <.001

Conc 2 128575.6 64287.8 255.93 <.001

Genotype.Conc 276 (2) 116023.2 420.4 1.67 <.001

Residual 1761 (514) 442351.4 251.2

Total 2183 (516) 781392.0

Supplementary Table 13) Salt germination screen comparing coleoptile emergence times, with line/cultivar (denoted as “Genotype”) and NaCl concentration (denoted as “Conc”) as the variates. Using the replicate (denoted as “Rep stratum”) as the blocking factor.

Source of variation d.f. (m.v.) s.s. m.s. v.r. F pr.

Rep stratum 5 66115.3 13223.1 56.36

Rep.*Units* stratum

Genotype 139 190811.2 1372.7 5.85 <.001

Conc 2 773960.2 386980.1 1649.43 <.001

Genotype.Conc 259 (19) 156128.9 602.8 2.57 <.001

Residual 1313 (962) 308048.8 234.6

Total 1718 (981) 942140.8

Supplementary Table 14) Salt germination screen comparing root length over time after a Log_10_ transformation, with line/cultivar sub-groups (Bere, other landraces, and elites; denoted as “Type”) and NaCl concentration (denoted as “Conc”) as the variates and day as the time factor.

Source of variation d.f. (m.v.) s.s. m.s. v.r. F pr.

ID stratum

Type 2 6.71340 3.35670 12.04 <.001

Conc 2 1034.34747 517.17373 1855.33 <.001

Type.Conc 4 1.63397 0.40849 1.47 0.210

Residual 2176 (515) 606.55973 0.27875 14.39

ID.Day stratum

d.f. correction factor 0.5154

Day 4 1217.27448 304.31862 15712.04

Day.Type 8 3.20855 0.40107 20.71 <.001

Day.Conc 8 79.93952 9.99244 515.91

Day.Type.Conc 16 0.65015 0.04063 2.10 0.031

Residual 8696 (2068) 168.42846 0.01937

Total 10916 (2583) 2687.68937

Supplementary Table 15) Salt germination screen comparing root length over time after a Log_10_ transformation, with line/cultivar (denoted as “Genotype”) and NaCl concentration (denoted as “Conc”) as the variates and day as the time factor.

Source of variation d.f. (m.v.) s.s. m.s. v.r. F pr.

ID stratum

Genotype 139 181.31307 1.30441 5.64 <.001

Conc 2 1022.87049 511.43524 2211.79 <.001

Genotype.Conc 276 (2) 106.64807 0.38641 1.67 <.001

Residual 1767 (513) 408.58674 0.23123 13.57

ID.Day stratum

d.f. correction factor 0.5437

Day 4 1212.31803 303.07951 17784.19

Day.Genotype 556 38.77135 0.06973 4.09 <.001

Day.Conc 8 80.70290 10.08786 591.94

Day.Genotype.Conc 1104 (8) 31.64902 0.02867 1.68 <.001

Residual 7060 (2060) 120.31704 0.01704

Total 10916 (2583) 2687.68937
